# Treatment of refractory low back pain due to arthrosis of the lumbar spine with or without spondylolisthesis using anterior lumbar interbody fusion (ALIF)

**DOI:** 10.1590/1806-9282.2023D702

**Published:** 2024-02-26

**Authors:** Adriano Anzai, Haroldo Katayama, Ighor Alexander Zamuner Spir, Mary Martins Nery, Mauricio Anhesini, Oswaldo Silvestrini Tiezzi, Patricia Rodrigues Naufal Spir, Pericles Otani, Wanderley Marques Bernardo

**Affiliations:** 1 Guidelines Program of the Brazilian Medical Association - São Paulo (SP), Brazil.

## INTRODUCTION

Low back pain due to osteoarthritis is among the most common causes of medical consultations, and in approximately 85% of cases, the origin of back pain is unknown. Osteoarthritis is a degenerative and progressive musculoskeletal disorder, a common condition involving joint surfaces, which can evolve into a debilitating condition due to pain and restricted movement.

Osteoarthritis is a multifaceted, progressive, irreversible condition that can progress to radiculopathy, myelopathy, spinal stenosis, degenerative spondylolisthesis, and hernias. Its etiology has not yet been fully established and can be attributed to multiple factors, including aging, living conditions, biomechanical load, and various molecular and genetic factors. At the cellular level, there are a reduction in the number of active cells, depletion of the extracellular matrix, an altered phenotype of normal disc cells, and the presence of cytokines and pro-inflammatory mediators such as interleukin (IL) 1β, IL-6, and IL-8, in association with degeneration.

Spondylolysis is a phenomenon that can be present, such as an anatomical defect or an interarticular fracture of the vertebral arch, which can progress to spondylolisthesis, defined as an anterior displacement of the vertebral body in reference to the adjacent vertebral bodies, and a dysplastic process that results in rounding anterior and superior of the S1 vertebrae. This rounding allows the L5 vertebrae to slide anteriorly onto the S1 vertebrae.

Although most cases of pain (low back pain) related to spinal arthritis are self-limited, requiring only conservative therapy, there are situations in which clinical control is difficult (refractoriness), and surgical treatment may be indicated and performed through lumbar interbody fusion (arthrodesis) via a posterior approach (PLIF), an anterior approach (ALIF), an oblique lateral approach (OLIF), or a transforaminal approach (TLIF).

## OBJECTIVE

The objective of this study was to systematically review the literature looking for comparative studies between the ALIF versus PLIF or TLIF or OLIF techniques in the surgical treatment of patients with refractory low back pain due to osteoarthritis.

## METHODOLOGY

In the methodology, we will express the clinical question, the structured question (PICO), study’s eligibility criteria, sources of information consulted and search strategies used, critical evaluation method (risk of bias) and quality of evidence, data to be extracted, and measures to be used to express results and the method of analysis.

## CLINICAL QUESTION

In patients with lumbar osteoarthritis (with or without spondylolisthesis) and pain refractory to conservative treatment, is surgery using the ALIF technique more effective and safe when compared with that using the PLIF, TLIF, or OLIF techniques?

## STRUCTURED QUESTION

P- patients with osteoarthritis and refractory low back pain (with or without spondylolisthesis);
I- ALIF technique;
C- TLIF or OLIF or PLIF techniques;
O- pain control, functional efficacy, or safety.

## SOURCES OF INFORMATION CONSULTED AND SEARCH STRATEGIES

The sources consulted were MEDLINE, EMBASE, ClinicalTrials, Scholar, and a manual search of the references of the included references.

The following strategies were used:

#1 (Previous lumbar interbody fusion OR ALIF);
#2 (Arthrodesis OR Arthrodeses OR Spinal Fusion OR Spinal Fusions OR Spondylodesis OR Spondylodeses OR Spondylosyndesis OR Spondylosyndeses) AND (Lordosis OR Lumbar Vertebrae OR Spondylolisthesis OR Lumbosacral Region);
#3 (#1 AND (comparative study) OR (((clinical[ Title/Abstract] AND trial[Title/Abstract]) OR clinical trials as topic[ MeSH Terms] OR clinical trial[Publication Type] OR random*[Title/Abstract] OR random allocation[MeSH Terms] OR therapeutic use[ MeSH Subheading]));
#4 (#2 AND Random*);
#5 (#3 OR #4).

## ELIGIBILITY CRITERIA

- Structured question elements;
- Comparative studies (observational or experimental);
- No period restriction;
- Languages: Portuguese, Spanish, and English;
- Full text or abstracts with data;
- Studies with data (continuous or categorical variables) available.

## RISK OF BIASES AND QUALITY OF EVIDENCE

The risk-of-bias items to be assessed will be in the case of:

- Randomized trials: randomization, blindfolded allocation, double blinding, blinding of evaluators, losses, prognostic characteristics, analyzed outcomes, sample calculation, early interruption, and analysis by intention to treat.
- Non-randomized clinical trials or observational cohort studies: confounding, selection, classification, interventions, protocol deviations, losses, outcomes, and results presented.

The quality of evidence will be classified as very low, low, and high when extrapolated directly from the risk of bias (if it is not possible to express the results through meta-analysis). If the results are expressed by meta-analysis, the quality items to be considered in assessing the quality of the evidence, classified by risk as very serious, serious, or not serious, will be type of study design, risk of bias, imprecision, indirect evidence, inconsistency, publication bias, magnitude of effect, dose-response, and confounding. The quality of the evidence can be classified as very low, low, moderate, and high.

## EXTRACTED DATA

The extracted data include name of the first author, year of publication, patient characteristics, intervention characteristics, analyzed outcomes, and follow-up time.

## OUTCOME MEASURES AND ANALYSIS

For categorical variables, absolute numbers, percentage, absolute risk, a reduction or an increase in risk, number needed to treat (NNT), or number needed to harm (NNH) will be used. For continuous variables, means with standard deviation and difference in means will be used. The confidence level will be 95% (95%CI). The goal is to aggregate the results of two or more studies for common outcomes.

If it is possible to aggregate the results of one or more included studies in relation to one or more common outcomes, a meta-analysis will be carried out as a way of expressing and supporting the conclusions. The inconsistency (heterogeneity) of the analysis will be evaluated by I^2^, varying between 0 and 100%. The random-effects model is used if I^2^>50% and the fixed-effects model if I^2^≤50%. To assess possible publication bias, the Egger test will be applied and visually expressed by the “funnel plot” (asymmetry).

## RESULTS

**Figure 1.**
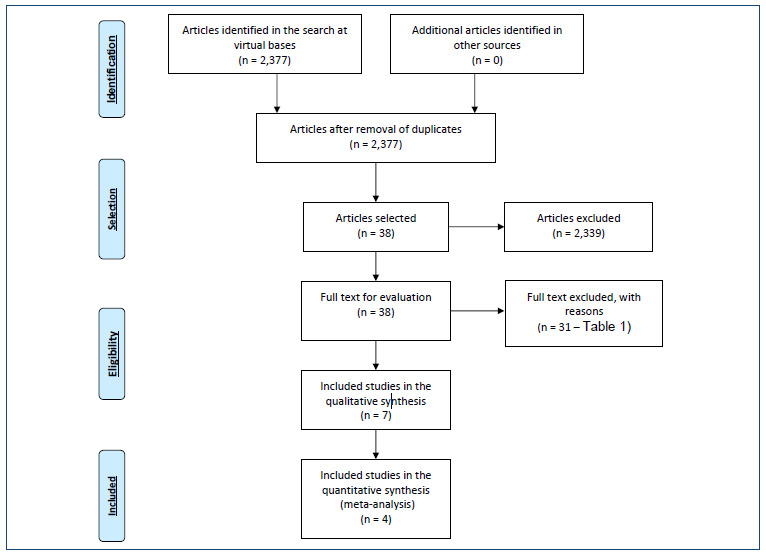
Diagram of retrieved and selected evidence (anterior lumbar interbody fusion).

In the search for evidence, a total of 2,377 studies were retrieved, 2,346 of which were inMEDLINE, 14 in EMBASE, 12 in the ClinicalTrials database, and 5 in Scholar. Probably meeting the eligibility criteria, 38 works were initially selected, which, by reading their full texts, allowed the final selection of seven publications^1^ ^,^ ^2^ ^,^ ^3^ ^,^ ^4^ ^,^ ^5^ ^,^ ^6^ ^,^ ^7^ to support this evaluation (Table 1 and Figure 1). The reasons for exclusion are given in Table 1.

**Table 1.** Description of included studies.

First Author/Year	Design	Population	Intervention	Comparison	Outcome	Follow-up
Tung et al.^1^	Retrospective cohort	Patients with lower back pain or sciatica that did not respond to conservative treatment for over 6 months due to degenerative spinal conditions; (2) lumbar interbody fusion with no more than four index levels fused (N: 348)	ALIF (N: 69)	OLIF (N: 101), TLIF (N: 178)	Health-related quality of life (HRQoL), including theODI, the EuroQol-5-dimension score (EQ-5D), the VAS of pain for total symptoms (VASTotal), for symptoms in the affected leg (VAS-Leg), and for symptoms in the back (VAS-Back), success	1 month, 3 months, 6 months, 1 year, and 2 years
Jacob et al.^2^	Retrospective review cohorts	Inclusion criteria permitted the study of patients who underwent primary, elective, single-level TLIF and ALIF procedures (N: 405)	Patients undergoing ALIF were positioned in a supine fashion on a flat table. The indicated disc level was preoperatively identified via fluoroscopy, and an anterior paramedian approach was performed (N: 59)	All MIS-TLIF procedures were performed using the Wiltse technique through a paramedian (4.5-cm skin incision lateral to midline) approach under fluoroscopic guidance (N: 346)	PROMIS-PF, VAS back and leg ODI, SF-12 PCS.	6 weeks, 12 weeks, 6 months, 1 year, and 2 years
Kuang et al.^3^	Retrospective review	Patients inclusion: (1) back and leg pain unresponsive to conservative treatment; (2) aged between 18 and 65 years; (3) noncalcified lumbar disc herniation compressing neuronal structures, as confirmed by magnetic resonance imaging (MRI); (4) patients with instable spine (N: 82)	MO-ALIF-patient positioned supine. A 3- to 5-cm transverse skin incision parallel to the affected disc level was made on the lateral wall of abdomen. Followed by blunt dissection of abdominal muscles, the peritoneal content was mobilized inward (N: 42)	TLIF-patients were placed in prone and inserted with pedicle screws. Pedicle screws were distracted, and then a discectomy was performed. A PEEK cage was placed after endplate preparation (N: 40)	ODI VAS back and leg	3, 12, and 24 months
Lee et al.^4^	Retrospective review	Patients inclusion-1) diagnosed as L4-5 single-level spondylolisthesis; 2) no ASD preoperatively; and 3) a minimum follow-up duration of 12 months.	ALIF-left-sided retroperitoneal approach was undertaken through a 5-cm paramedian incision in mini-ALIF fashion. After removal of the disc material and posterior anulus fibrosus (N: 27)	PLIF-standard midline exposure was undertaken. Under the microscope, bilateral or unilateral laminotomies with partial or complete facetectomies and foraminotomies .(N: 31)	ODI VAS back and leg	12 months
Lee et al.^5^	Retrospective review	Patients aged 20-80 years had severe lower back pain as a chief complaint, and leg pain or neurogenic intermittent claudication collaterally. diagnosed with spondylolytic spondylolisthesis on, with degenerative lumbar spinal stenosis on L5-S1 (N: 77)	ALIF was performed in patients who complained primarily of lower back pain, rather than leg pain or neurogenic intermittent claudication (N: 26)	Patients who primarily complained of single leg pain were treated with TLIF (N: 21). Patients who complained of low back pain, leg pain, and neurogenic intermittent claudication were treated with PLIF and pedicle screw fixation (N: 30).	VAS back	21.6 months (range, 12-84 months)
Kim et al.^6^	Retrospective review	Patients aged 18-65 years were the presence of single-level low-grade isthmic spondylolisthesis, chronic and persistent radiculopathy despite conservative treatment, progressive neurological deficits, persistent and unremitting lower-back pain for more than 6 months, loss of quality of life because of neurological claudication (N: 94)	All ALIF procedures were performed using the mini-laparotomic retroperitoneal approach, as previously described. After discectomy, a large, wedge-shaped, lordotic cage (N: 48)	TLIF-The surgery was performed through a mini-open fashion with expandable working tubes; alternatively, the surgery was performed in a minimally invasive fashion using nonexpandable working tubes and the percutaneous N46	VAS; score range: 0-10 ODI	24 months
Madan et al.^7^	Prospective study	Patients aged 24-67 years-severe symptoms of low back pain not responding to medication, rehabilitation, and conservative treatment present for at least 2 years (N: 74)	ALIF-The operation was performed through a direct anterior transperitoneal approach for L5-S1 and a standard anterolateral retroperitoneal approach for the other lumbar levels (N: 39)	PLIF-In the circumferential fusion group with PLIF, the approach was midline posterior. Laminectomy and facetectomy were done (N: 35)	VAS; ODI	24 months

### Description of included studies (Table 1)

A total of 1,138 patients with low back pain refractory to conservative treatment, in the presence of lumbar spine arthrosis with or without spondylolisthesis, were studied. Of them, 310 patients underwent the ALIF technique, compared with 631, 101, and 96 patients who underwent the TLIF, OLIF, and PLIF techniques, respectively.

The possible outcomes to be considered to support the effectiveness analysis were the Oswestry Disability Index (ODI) and pain (VAS-visual analog scale), since these outcomes were evaluated by all included studies, differing only by the length of follow-up (6 months, 12 months, or 24 months). The ODI was applied through a questionnaire, where the final score ranged from 0 to 100. A score of 0-20 reflects minimal disability, 21-40 moderate disability, 41-60 severe disability, 61-80 cripple, and 81-100 bedridden. Regarding pain measured by the VAS, the score ranged from 0 to 10.

## DIAGRAM OF RETRIEVED AND SELECTED EVIDENCE (FIGURE 1 - ALIF)

### risk of bias (Table 2)

**Table 2.** Risk of bias.

Studies	Confounding	Selection	Intervention	Deviation	Losses	Outcomes	Results
Tung et al.^1^							
Jacob et al.^2^							
Kim et al.^6^							
Kuang et al.^3^							
Lee et al.^4^							
Lee et al.^5^							
Madan et al.^7^							
Low risk of bias	Without Information	High risk of bias

The overall risk of bias is high (all studies aggregated), due to limitations of confounding items, classification of interventions, and patient selection.

### Analysis results (Table 3)

**Table 3.** Analysis results.

Table of results																	
STUDIES	ODI (6 M) (Median) (SD)			ODI (1 Year)				ODI (2 Years)				VAS back pain (1 YEAR)			VAS back pain (2 Years)		
	ALIF	OLIF	TLIF	ALIF	OLIF	TLIF	PLIF	ALIF	OLIF	TLIF	PLIF	ALIF	TLIF	PLIF	ALIF	TLIF	PLIF
Tung et al.^1^	26.5 (16.3) (N: 69)	31.8 (14.8) (N:101)	35 (13.9) (N: 178)	26.5 (16.3) (N: 69)	31.8 (14.8) (N: 101)	35 (13.9) (N: 178)		26.5 (16.3) (N: 69)	31.8 (14.8) (N:101)	35 (13.9) (N:178)							
Jacob et al.^2^	24.5 (19.9) (N59)		22.8 (19.7) (N346)	22.0 (28.8) (N59)		24.5 (22.0) (N346)		32.0 (21.2) (N59)		23.9 (28.7) (N346)		2.6 (3.2) (N59)	3.3 (2.6) (N346)				
Kim et al.^6^								23.2 (18.1) (N48)		14.4 (15.9) (N46)					2.9 (2.4) (N48)	2.3 (2.6) (N46)	
Kuang et al.^3^				24.2 (7.5) (N42)		25.0 (6.9) (N40)		24.9 (7.1) (N42)		24.4 (7.7) (N40)		2.5 (1.2) (N42)	2.7 (1.7) (N40)		2.3 (1.4) (N42)	2.6 (1.8) (N40)	
Lee et al.^4^				25 (18.5) (N27)			20 (14.2) (N31)					2.2 (2.3) (N27)		2.7 (2.4) (N31)			
Lee et al.^5^															2.73 (1.61) (N26)	1.52 (1.20) (N21)	1.97 (1.42) (N30)
Madan et al.^7^								32.9 (20.5) (N39)			30.5 (21.5) (N35)						

### ODI Outcome (6 months, 12 months, and 24 months)

The ODI for pain in the leg and back outcome was included in the analysis of four studies^1^ ^,^ ^2^ ^,^ ^3^ ^,^ ^4^: in the 6-month follow-up, two studies^1^ ^,^ ^2^ (1 comparing with OLIF^1^ and 2 comparing with TLIF^1^ ^,^ ^2^) and in the 12-month follow-up, four studies^1^ ^,^ ^2^ ^,^ ^3^ ^,^ ^4^ (1 comparing with OLIF^1^, 3 comparing with TLIF^1^ ^,^ ^2^ ^,^ ^3^, and 1 comparing with PLIF^4^). The results of the remaining three included studies^5^ ^,^ ^6^ ^,^ ^7^ are only described (Table 3) and will not be considered in the conclusions of this evaluation. It was not possible to evaluate safety outcomes due to lack of data.

### 1a. Follow-up time of 6 months (Figure 2)^*1*^ ^*,*^ ^*2*^

**Figure 2.**
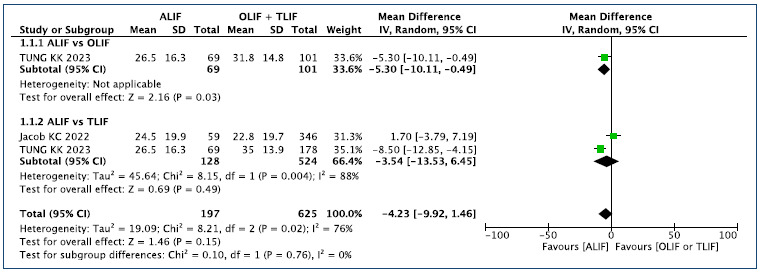
ODI Outcome - Follow-up time of 6 months.

This analysis includes two comparisons of ALIF versus OLIF and TLIF. When compared with OLIF (N: 101), the ALIF technique (N: 69) reduces the ODI by 5% [-5.3 95%CI (-0.49 to -10.1)] of the total 100 points (26.5 versus 31.8). In comparison with TLIF, there is no difference in the final ODI. In the global analysis, by comparing the result of ALIF technique with the aggregated results of OLIF and TLIF, there is no difference in the ODI obtained at 6 months of follow-up.

### 1b. Follow-up time of 12 months (Figure 3)^*1*^ ^*,*^ ^*2*^ ^*,*^ ^*3*^ ^*,*^ ^*4*^

**Figure 3.**
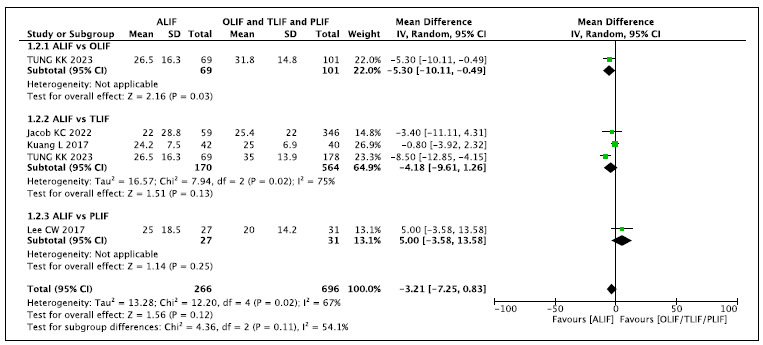
ODI Outcome - Follow-up time of 12 months.

This analysis includes three comparisons of ALIF versus OLIF, TLIF, and PLIF. When compared with OLIF (N: 101), the ALIF technique (N: 69) reduces the ODI by 5% [-5.3 95%CI (-0.49 to -10.1)] of the total 100 points (26.5 versus 31.8). In comparison with TLIF and PLIF, there is no difference in the final ODI. In the global analysis, by comparing the result of the ALIF technique with the aggregated results of OLIF, TLIF, and PLIF, there is no difference in the ODI obtained at 12 months of follow-up.

### 1c. Follow-up time of 24 months (Figure 4)^*1*^ ^*,*^ ^*2*^ ^*,*^ ^*3*^ ^*,*^ ^*6*^ ^*,*^ ^*7*^

**Figure 4.**
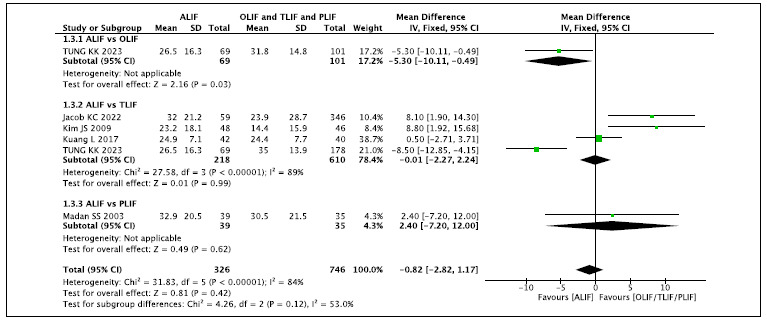
ODI Outcome - Follow-up time of 24 months.

This analysis includes three comparisons of ALIF versus OLIF, TLIF, and PLIF. When compared with OLIF (N: 101), the ALIF technique (N: 69) reduces the ODI by 5% [-5.3 95%CI (-0.49 to -10.1)] of the total 100 points (26.5 versus 31.8). Compared with TLIF and PLIF, there is no difference in the final ODI. In the global analysis, by comparing the result of ALIF technique with the aggregated results of OLIF, TLIF, and PLIF, there is no difference in the ODI obtained at 24 months of follow-up.

### Pain outcome (VAS) (12 months and 24 months)

### 2a. Follow-up time of 12 months (Figure 5)^*2*^ ^*,*^ ^*3*^ ^*,*^ ^*4*^

**Figure 5.**
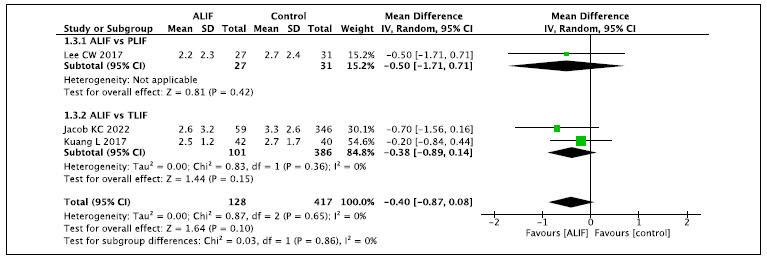
Pain outcome (VAS) - Follow-up time of 12 months.

This analysis includes two comparisons of ALIF versus PLIF and TLIF. When compared with PLIF (N: 31), the ALIF technique (N: 27) does not reduce pain (VAS). Compared with TLIF (N: 386), and in the global analysis, there is also no difference in the final pain (VAS) at the 12-month follow-up.

### 2b. Follow-up time of 24 months (Figure 6)^*3*^ ^*,*^ ^*5*^ ^*,*^ ^*6*^

**Figure 6.**
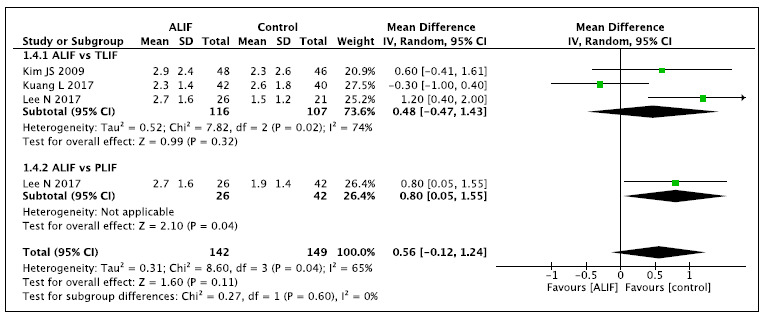
Pain outcome (VAS) - Follow-up time of 24 months.

This analysis also includes two comparisons of ALIF versus PLIF and TLIF. When compared with TLIF (N: 116), the ALIF technique (N: 107) does not reduce pain (VAS). Compared with PLIF (N: 42), the ALIF technique (N: 26) increases pain (VAS) by 8% [+0.8 95%CI (+0.05 to +1.55)] of the total 10 points (2.7 versus 1.9),. In the global analysis, there is no difference in pain between the comparisons at the 24-month follow-up.

### Quality of evidence (Table 4) 

**Table 4.** Anterior lumbar interbody fusion for spondylolisthesis.

Certainty assessment							No. of patients		Effect		Certainty	Importance
No. of studies	Study design	Risk of bias	Inconsistency	Indirect evidence	Imprecision	Other considerations	ALIF	OLIF or TLIF	Relative (95%CI)	Absolute (95%CI)		
ODI 6m												
2	Observational study	Serious^a^	Not serious	Not serious	Not serious	None	197	625	-	MD **4.23 lower** (9.92 lower to 1.46 higher)	⨁◯◯◯ very low	
ODI 6m-ALIF versus OLIF												
1	Observational study	Serious^a^	Very serious^b^	N ot serious	Very serious^c^	None	69	101	-	MD **5.3 lower** (10.11 lower to 0.49 lower)	⨁◯◯◯ very low	
ODI 6m-ALIF versus TLIF												
2	Observational study	Serious^a^	Very serious^b^	Not serious	Serious^d^	None	128	524	-	MD **3.54 lower** (13.53 lower to 6.45 higher)	⨁◯◯◯ very low	
ODI 12m												
4	Observational study	Serious^a^	Not serious	Not serious	Not serious	None	266	696	-	MD **3.21 lower** (7.25 lower to 0.83 higher)	⨁◯◯◯ very low	
ODI 12m-ALIF versus OLIF												
1	Observational study	Serious^a^	Serious^e^	Not serious	Serious^d^	None	69	101	-	MD **5.3 lower** (10.11 lower to 0.49 lower)	⨁◯◯◯ very low	
ODI 12m-ALIF versus TLIF												
3	Observational study	Serious^a^	Not serious	Not serious	Serious^d^	None	170	564	-	MD **4.18 lower** (9.61 lower to 1.26 higher)	⨁◯◯◯ very low	
ODI 12m-ALIF versus PLIF												
1	Observational study	Not serious	Serious^e^	Not serious	Serious^d^	None	27	31	-	MD 5 higher (3.58 lower to 13.58 higher)	⨁◯◯◯ very low	
ODI 24m												
5	Observational study	Serious^a^	Very serious^b^	Not serious	Very serious^c^	None	326	746	-	MD 0.82 lower (2.82 lower to 1.17 higher)	⨁◯◯◯ very low	
ODI 24m-ALIF versus OLIF												
1	Observational study	Serious^a^	Not serious	Not serious	Not serious	None	69	101	-	MD 5.3 lower (10.11 lower to 0.49 lower)	⨁◯◯◯ very low	
ODI 24m-ALIF versus TLIF												
4	Observational study	Serious^a^	Serious^e^	not serious	Very serious^c^	None	218	610	-	MD 0.01 lower (2.27 lower to 2.24 higher)	⨁◯◯◯ very low	
ODI 24m-ALIF versus PLIF												
1	Observational study	Serious^a^	Not serious	Not serious	Very serious^c^	None	39	35	-	MD 2.4 higher (7.2 lower to 12 higher)	⨁◯◯◯ very low	
Pain (VAS) 1 year												
3	Observational study	Serious^a^	Not serious	Not serious	Serious^d^	None	128	417	-	MD 0.4 lower (0.87 lower to 0.08 higher)	⨁◯◯◯ very low	
Pain (VAS) 1 year-ALIF versus PLIF												
1	Observational study	Serious^a^	Not serious	Not serious	Very serious^c^	None	27	31	-	MD 0.5 lower (1.71 lower to 0.71 higher)	⨁◯◯◯ very low	
Pain (VAS) 1 year-ALIF versus TLIF												
2	Observational study	Serious^a^	Not serious	Not serious	Serious^d^	None	101	386	-	MD 0.38 lower (0.89 lower to 0.14 higher)	⨁◯◯◯ very low	
Pain (VAS) 2 years												
3	Observational study	Serious^a^	Not serious	Not serious	Not serious	None	142	149	-	MD 0.56 higher (0.12 lower to 1.24 higher)	⨁◯◯◯ very low	
Pain (VAS) 2 ANOS-ALIF versus TLIF												
3	Observational study	Serious^a^	Serious^e^	Not serious	Very serious^c^	None	116	107	-	MD 0.48 higher (0.47 lower to 1.43 higher)	⨁◯◯◯ very low	
DOR (VAS) 2 ANOS-ALIF versus PLIF												
1	Observational study	Serious^a^	Not serious	Not serious	Not serious	None	26	42	-	MD 0.8 higher (0.05 higher to 1.55 higher)	⨁◯◯◯ very low	

CI: confidence interval; MD: mean difference. ^a^Problems in the confounding, selection and in the intervention classification. ^b^Heterogeneity higher of 75%. ^c^CI very large. ^d^CI large. ^e^Heterogeneity between 50 and 75%.

The quality of evidence in all analyses is very low, with the biggest limitations being observational study design in the absence of randomized clinical trials, inconsistency (high heterogeneity), and imprecision (small size and effect differences of the samples studied).

## SUMMARY OF THE EVIDENCE

In patients with osteoarthritis and low back pain refractory to conventional treatment, there is very low quality evidence evaluating the ALIF technique in comparison with the OLIF, TLIF, or PLIF techniques. Furthermore, there is no measurement of outcomes common to the few studies available, which would allow for an aggregated analysis of results, whether in terms of efficacy (only the ODI) or safety. In relation to the outcomes measured by the ODI and VAS for pain, there is no difference (no reduction) in the results in the 6-, 12-, or 24-month follow-ups, which allows us to recommend this technique in the treatment of these patients, especially if we consider the comparison to the posterior access currently in use (PLIF).
